# Laser-Induced Nanocarbon Films Enable Optical Sensor Based on Combined Photothermal and Piezoresistive Effect

**DOI:** 10.3390/polym18121533

**Published:** 2026-06-19

**Authors:** Yanbo Yao, Jingwen Yao, Tao Liu

**Affiliations:** 1 School of Materials and Packaging Engineering, Fujian Polytechnic Normal University, Fuqing 350300, China; yaoyb@fpnu.edu.cn; 2 College of Chemistry, Chemical Engineering and Materials Science, Soochow University, Suzhou 215123, China; yaojingweno@163.com

**Keywords:** laser-induced nanocarbon films, optical sensor, photothermal effect, piezoresistive effect, broadband spectral response

## Abstract

This work presents an enhanced photomechanical optical sensor inspired by our previously reported bio-inspired uncooled infrared detector. Performance improvement is achieved by strengthening the interfacial bond between the photothermal dendrite—polydopamine nanoparticle (PDA NP)/polydimethylsiloxane (PDMS) composite—and the piezoresistive laser-induced nanocarbon film, with a flexible PDMS substrate that provides both thermal insulation and mechanical stability. The resulting sensor exhibits a responsivity of 51.6 W^−1^ under 808 nm irradiation, an order-of-magnitude enhancement over the unmodified device. Wavelength-dependent characterization (455–1550 nm) shows responsivity decreasing from 93.1 W^−1^ at 455 nm to 14.4 W^−1^ at 1550 nm, with response times on the order of seconds across this range. Extending this trend into the longer-wavelength region of blackbody radiation, the mechanism transitions to a predominantly bolometric mode. The device also demonstrates stable detection of several hundred microwatts and robust durability at 455 nm. These results validate interface engineering strategy as a viable pathway toward high-performance uncooled optical detection, advancing bio-inspired detectors from functional mimicry toward an application-ready platform. These findings confirm PDA NPs as effective photothermal converters primarily at shorter wavelengths, while the wavelength-dependent response suggests future tailoring of spectral sensitivity using long-wavelength-absorbing materials.

## 1. Introduction

Infrared (IR) sensing technology has found significant applications in both military and civilian fields, including industrial equipment monitoring, security surveillance, disaster relief, remote sensing, traffic management, and medical diagnostics [1,2,3]. However, traditional thermal-based IR sensing technologies, which rely on rigid and costly devices such as thermocouples [4,5] and pyroelectric detectors [6,7], are insufficient to meet the growing demands of emerging applications like electronic skins [8] and soft robotics [9]. This limitation has accordingly driven widespread research interest in developing low-cost, miniaturized flexible IR sensing systems. In this context, certain pyrophilous beetles, such as *Melanophila acuminata*, have evolved a photomechanical IR sensing mechanism that achieves exceptional sensitivity through a synergy of thermal expansion and thermo-mechanical effect [10,11,12,13]. This biological blueprint offers a compelling pathway for developing structural simple, low-cost and uncooled IR sensors [14,15,16,17,18], thereby presenting a viable alternative to incumbent technologies while enabling compatibility with emerging flexible and printable electronics [19]. Building on this paradigm, our previous work established a bio-inspired IR sensor architecture that replicates the beetle’s stress-concentrated mechanotransduction while replacing its fluidic system with solid-state electromechanical coupling [20]. These design choices yield a compact, electrically integrable, and scalable platform well suited for applications where cost, portability, and manufacturability are as critical as sensitivity [21,22,23,24]. Finite element analysis of the device revealed two distinct operational regimes—bolometric and photomechanical—providing a theoretical basis for understanding and improving its sensing behavior. However, in that initial design, the piezoresistive sensing element—a laser-fabricated nanocarbon film [25]—and the photothermal actuator—a polydopamine nanoparticle (PDA NP)-doped polydimethylsiloxane (PDMS) elastomer—were only in physical contact. This configuration prevented effective transfer of photothermally induced deformation to the piezoresistive film. Consequently, the measured photomechanical response fell substantially below theoretical predictions.

**Figure 1:**
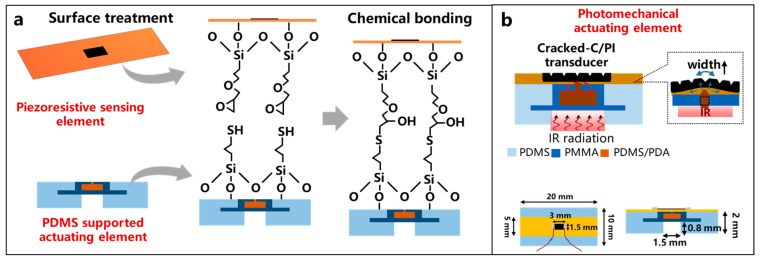
(**a**) Schematic of the interfacial bonding strategy through O_2_ plasma treatment followed by silane coupling, covalently bonding the PDMS-supported photothermal dendrite to the laser-induced nanocarbon piezoresistive film. (**b**) Working principle of the optical sensor in photomechanical sensing mode enabled by the enhanced mechanical coupling at the integrated photothermal–piezoresistive interface, achieved through the process in (**a**). The inset shows a structural schematic of the sensor.

Herein, this work addresses this interfacial limitation by transitioning from simple contact to an integrated, bonded interface. As illustrated in Figure 1a, O_2_ plasma treatment followed by silane coupling is employed to covalently bond the photothermal dendrite to the laser-induced nanocarbon piezoresistive film, ensuring efficient transmission of photothermally induced deformation to the piezoresistive membrane. To assess its application potential, we evaluate the sensor’s broadband spectral response across the visible to near-infrared range (455–1550 nm) and under blackbody radiation, revealing a distinct wavelength-dependent responsivity with the sensing mechanism transitioning from photomechanical to bolometric mode at longer wavelengths. The enhanced interfacial bonding yields substantial improvements in device performance, including high sensitivity, stable several-hundred-microwatt detection, fast response, and robust durability over extended cycling—attributes validated particularly in the shorter-wavelength regime. Collectively, these results validate the interface engineering strategy as a viable pathway toward high-performance uncooled optical detection, advancing the bioinspired detector from functional mimicry toward an application-ready device. Furthermore, this observed wavelength-dependent behavior highlights the potential for tailoring spectral sensitivity through future engineering of photothermal materials.

## 2. Experimental and Methods

### 2.1. Fabrication of the Sensing Element

The fabrication process of the optical sensor in this work largely follows the previously reported procedures [20], which include: (i) fabrication of the photothermal/photomechanical actuating element by preparing PDA NP-doped PDMS composites (with a PDA concentration of 1.5 wt.%), empirically selected based on prior systematic studies showing that higher concentrations cause PDA NP agglomeration and reduce photothermal efficiency, and filling a two-stage cylindrical PMMA cavity—featuring a perforation at its upper end for localized stress concentration—with the nanocomposite; and (ii) preparation of the C/polyimide (PI) piezoresistive film as the mechanical deformation sensing unit. A structural schematic of the sensor is shown in Figure 1b. Specifically, to achieve high piezoresistive sensitivity, aligned microcrack arrays were introduced into the laser-induced C/PI films through repeated uniaxial stretching, following the protocol established in our previous work [25]. The strain-training process was conducted using a dynamic mechanical analysis (DMA, T800, TA Instruments, New Castle, DE, USA) instrument. During each cycle, the film was stretched at a constant rate of 1 N/min under programmed control, while the resistance change was monitored in situ by a digital multimeter (DMM, Keithley 3706A, Tektronix Co., Ltd., Shanghai, China). Each cycle consisted of loading until either the force reached 6 N or the resistance exceeded the 120 MΩ measurement range, followed by immediate load release.

Building on this established process, the present study introduces a comprehensive interfacial engineering strategy to improve optical sensing performance. This strategy consists of two integrated components: (1) flexible PDMS encapsulation of the photothermal/photomechanical actuating element, and (2) covalent interfacial bonding between the PDMS-supported actuating element and the piezoresistive film. The PDMS encapsulation procedure was carried out as follows. First, after preparing the photothermal/photomechanical actuating element, PDMS prepolymer was injected into a mold (20 × 10 × 1.6 mm^3^). Following degassing and thermal curing, the actuating element was placed onto the PDMS surface. A second injection of PDMS prepolymer was then performed until the liquid level reached the top of the PMMA shell, followed by another degassing and curing step. After demolding, a 1.5 mm × 1.5 mm window was opened at the bottom center of the PDMS block to allow direct laser irradiation onto the PMMA cavity surface. To achieve robust interfacial bonding between the PDMS-supported actuating element and the piezoresistive sensing film, as illustrated in Figure 1a, an O_2_ plasma treatment followed by silane coupling was applied. Both elements were first exposed to O_2_ plasma for 20 min to activate their surfaces. Subsequently, they were immersed separately in two different silane solutions: the actuating element in an isopropyl alcohol solution containing 1% (*v*/*v*) 3-mercaptopropyl trimethoxysilane (MPTMS, 97%, J&K Scientific, San Jose, CA, USA), and the sensing film in a methanol solution containing 1% (*v*/*v*) 3-glycidyloxypropyl trimethoxysilane (GPTMS, >97.0%, TCI, Shanghai, China). After 1 h of immersion, both components were rinsed with isopropyl alcohol and dried with nitrogen. The PI side of the sensing film was then carefully laminated onto the PDMS-supported actuating element, ensuring that the dendritic structure was centered over the carbon film. The assembly was weighted and left for 12 h to complete the silane coupling reaction, forming covalent bonds across the interface. Finally, silver electrodes were deposited onto the carbon film and wires were attached.

### 2.2. Characterization and Performance Evaluation of the Sensor

To evaluate the interfacial bond strength between PDMS and PI achieved through the plasma and silane-coupling treatment, a model assembly was prepared. A pure PDMS block (20 mm × 10 mm × 1.9 mm) and a pure PI film (6 mm × 20 mm) were bonded following the same surface treatment procedure described earlier, with an overlap area of 6 mm × 5 mm. The mechanical performance of the bonded assembly was then characterized under uniaxial tension using a universal testing machine (CMT4304, Zhuhai Sansi Taijie Electrical Equipment Co., Ltd., Zhuhai, China) at a crosshead speed of 1 mm/min. The response performance of the optical sensor was characterized using the measurement setup established in our previous work [20]. Both ends of the sensor were clamped onto a tension table and subjected to an initial strain of 0.7%. To evaluate the spectral response, the sensor was illuminated with single-wavelength laser sources spanning from 455 to 1550 nm. In addition, the sensor’s response to broadband infrared radiation was assessed using a blackbody radiation source at temperatures ranging from 300 °C to 500 °C. Detailed specifications of the laser and blackbody radiation sources are provided in Table 1. During laser or blackbody radiation, the resistance of the piezoresistive carbon film was recorded using the DMM at a sampling rate of 10 Hz.

## 3. Results and Discussion

**Figure 2:**
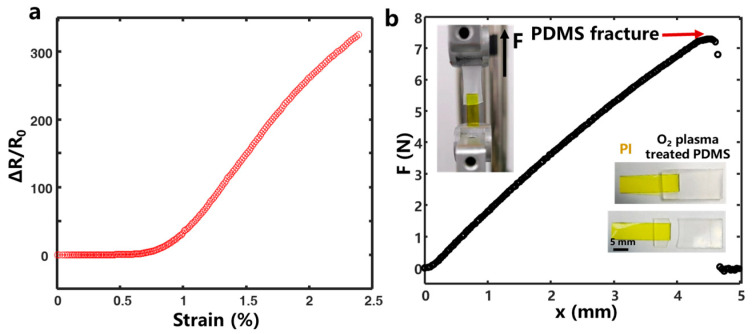
(**a**) Piezoresistive response of the strain-trained C/PI film after the piezoresistive behavior has stabilized, exhibiting a gauge factor of ~5200 at 0.7% strain. (**b**) Tensile test of the PDMS–PI interface after O_2_ plasma and silane treatment, with insets showing the sample before and after tensile failure.

To generate well-aligned microcrack arrays for high piezoresistive sensitivity [20,25], the C/PI composite films were subjected to repeated uniaxial stretching (strain training). After 10 stretching–releasing cycles, the piezoresistive response stabilized, indicating saturation of crack development—beyond this point, the intercrack spacing becomes smaller than the stress transfer length, preventing further crack formation. The stabilized relative resistance change as a function of applied strain is shown in Figure 2a. Consistent with previously reported behavior [20,25], the film exhibits relatively a nonlinear response, becoming increasingly sensitive with larger strain. To leverage this characteristic while maintaining a moderate pre-strain level, the film is pre-strained to 0.7% for sensor operation, where the piezoresistive gauge factor reaches ~5200. The enhanced PDMS–PI adhesion achieved through plasma treatment and chemical modification was verified by tensile tests (Figure 2b), in which failure occurred within the bulk PDMS while the bonded interface remained intact. The interfacial bonding strength was estimated to be at least 0.24 MPa. This result confirms the effectiveness of the combined O_2_ plasma treatment and silane coupling in strengthening the PDMS–PI interface, thereby presenting a reliable strategy for robustly integrating the photothermal actuating and piezoresistive sensing elements.

**Figure 3:**
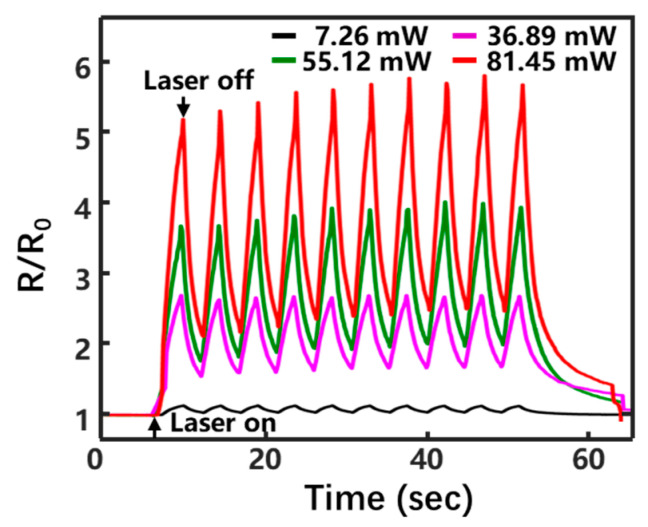
Relative resistance response of the sensor under 808 nm laser irradiation at varying optical power levels.

Building on the established 0.7% pre-strain and interface reinforcement strategies detailed above, the present sensor was designed and expected to operate predominantly in photomechanical regime. To evaluate its broadband spectral response, we characterized the device at three distinct wavelengths—455, 808, and 1550 nm—using pulsed laser irradiation. Figure 3 presents representative results obtained under 808 nm irradiation, measured at 0.1 Hz with a 50% duty cycle over ten on–off cycles at varying power levels while continuously monitoring resistance. The resistance synchronizes with the laser switching: it rises upon illumination and decays when the laser is turned off. This repeatable response confirms the dominance of the photomechanical mechanism. Upon illumination, the PDA NP-doped PDMS composite filling the PMMA cavity undergoes photothermal expansion. This expansion concentrates stress at the upper-end perforation and transmits the force to the overlying piezoresistive film. The resulting mechanical deformation widens the pre-existing microcracks within the film, leading to a measurable increase in resistance.

**Figure 4:**
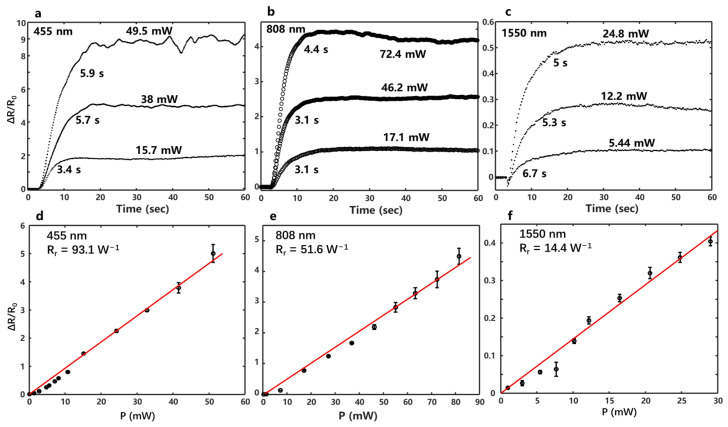
(**a**–**c**) Temporal photoresponse at three representative laser power levels and (**d**–**f**) power-dependent relative resistance change in the sensor under 455 nm (**a**,**d**), 808 nm (**b**,**e**), and 1550 nm (**c**,**f**) illumination. Linear fits in (**d**–**f**) are used to extract responsivity.

Building on the photomechanical response verified at 808 nm, we further characterized the sensor’s behavior across the 455–1550 nm wavelength range, with Figure 4a–c showing the time-dependent response at 455, 808, and 1550 nm, respectively, for three representative laser power levels. At all three wavelengths, the relative resistance increases rapidly upon laser illumination and gradually stabilizes. Using the time required for the resistance to reach 63.2% of its saturation value as the response time metric, the sensor exhibits response times of 5.9 s, 5.7 s, and 3.4 s at 455 nm for laser powers of 15.7 mW, 38 mW, and 49.5 mW, respectively. Under 808 nm and 1550 nm irradiation, the response times range from 3.1–4.4 s and 5.0–6.7 s, respectively, broadly consistent with those observed under 455 nm illumination. Figure 4d,e summarize the power-dependent response behavior across the 455–1550 nm wavelength range. At all wavelengths, the relative resistance change exhibits a good linear relationship with laser power, while the responsivity—defined as $R_{r}=d(∆R/R_{0})/dP$—gradually decreases with increasing wavelength. The sensor responsivity extracted from linear fitting reaches a maximum of 93.1 W^−1^ at 455 nm and a minimum of 14.4 W^−1^ at 1550 nm. This wavelength-dependent trend is consistent with the decreasing optical absorption coefficient of the PDA NP composite at longer wavelengths [20], which reduces photothermal conversion efficiency, leading to a smaller temperature rise, reduced thermal expansion, and consequently weaker bending stress on the sensing unit. Despite this general trend, the sensor exhibits a remarkable responsivity of 51.6 W^−1^ at 808 nm—an order-of-magnitude improvement over the non-optimized sensor (2.2 W^−1^) reported previously [20]. (For a comprehensive comparison with other organic material-based infrared sensors, see Table 1 of our previous work [20]). This order-of-magnitude gain in responsivity stems from an integrated interfacial engineering strategy. Specifically, the covalent bonding creates a chemical interface that enables efficient stress transfer from the expanding composite to the carbon film, while the PDMS encapsulation creates a mechanical interface that constrains the PI film to prevent stress relaxation. Additionally, the low thermal conductivity of PDMS plays a secondary role by thermally insulating the actuator to boost photothermal conversion. Together, these interfacial effects amplify the photomechanical response.

**Figure 5:**
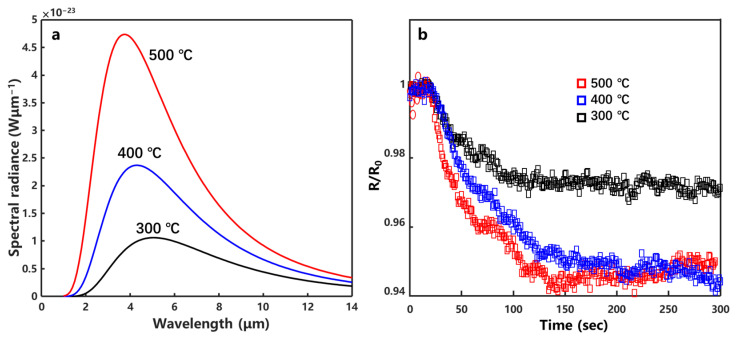
(**a**) Optical power spectrum at different set temperatures for the blackbody radiation source (DIAS Infrared Systems, CS500; aperture diameter = 60 mm; emissivity = 0.97) used to characterize the sensor’s response. (**b**) Evolution of the sensor’s relative resistance over irradiation time under the blackbody conditions shown in (**a**) for temperatures ranging from 300 °C to 500 °C.

Building on this wavelength-dependent trend, we anticipated that at even longer wavelengths—where PDA absorption becomes negligible—the dominant sensing mechanism may transition from photomechanical to bolometric. To investigate this, the sensor response was evaluated in the mid-infrared region using a broadband blackbody radiation source. Figure 5a shows the calculated optical power spectra at set temperatures of 300 °C, 400 °C, and 500 °C, where Figure 5b presents the corresponding relative resistance change as a function of irradiation time. As the blackbody temperature increases, the magnitude of the relative resistance change increases accordingly, reaching a maximum of −5.4% at 500 °C. The negative response indicates a bolometric operating mechanism, contrasting with the photomechanical dominance observed under shorter-wavelength laser excitation (455–1550 nm). In the mid-infrared region, the absorption coefficient of PDA NPs is expected to be too low to induce appreciable photothermal expansion in the actuating element, thereby suppressing mechanical deformation and allowing bolometric effects to prevail as the dominant sensing mode.

**Figure 6:**
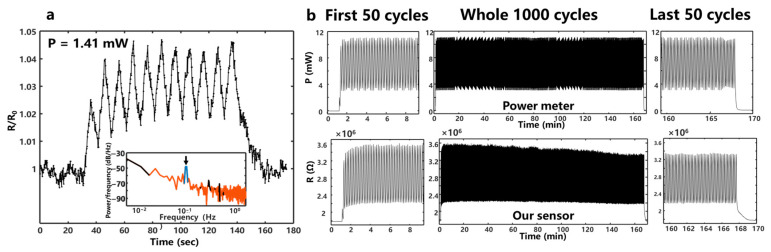
Sensor performance for low-power detection and long-term stability at 455 nm. (**a**) Resistance response under cyclic irradiation at a low power level of 1.41 mW. Inset: Frequency-domain periodogram of the time-domain signal shown in (**a**); the black curve represents the Fourier-transformed result, the blue peak indicates the dominant 0.1 Hz input signal, and the orange region/curve highlights the noise contribution. (**b**) Long-term stability evaluation over 1000 cycles of laser irradiation (0.1 Hz, 11 mW) with reference measurements from a commercial thermal power meter (S302C, Thorlabs).

Based on the wavelength-dependent responsivity characterized above—which showed the highest sensitivity at 455 nm—this wavelength was selected as a representative case for further evaluating the sensor’s low-power detection limit and long-term stability. Figure 6a shows the resistance response under cyclic irradiation at a relatively low power level of 1.41 mW. Time-domain measurements indicate that the sensor reliably detects laser power down to several hundred microwatts. To quantitatively assess the detection limit, frequency-domain periodogram analysis was performed on the time-domain data (inset of Figure 6a), from which the signal-to-noise ratio (SNR) was extracted to estimate the noise-equivalent power (NEP). The experimentally derived NEP was determined to be 0.9 mW/√Hz, consistent with time-domain estimates. Given the novelty of the materials and device architecture, this NEP value represents an initial result at the prototype stage. Further improvement in signal-to-noise ratio is needed to bridge the gap toward practical applications. Furthermore, to assess long-term stability, the sensor was subjected to 1000 cycles of laser irradiation (455 nm, 0.1 Hz, 11 mW). Figure 6b presents the sensor’s resistance response alongside reference measurements from a commercial thermal power meter (S302C, Thorlabs). Comparing the first and last 50 cycles, the baseline resistance (R_0_) increased by only 0.07% after 1000 cycles, while the laser-induced relative resistance change (ΔR/R_0_) decreased by 6.5%. This degradation is attributed to cumulative effects in the photothermal actuating element—such as creep, stress relaxation, or PDA NPs degradation under repeated heating–expansion cycles—rather than to instability of the laser-induced nanocarbon piezoresistive film, whose resistance drift remained negligible (0.07%). These findings suggest that future efforts should focus on optimizing the formulation and processing of the photothermal composite to enhance durability in optical sensing applications.

## 4. Conclusions

Inspired by the “fire beetle” infrared sensing organ, we previously developed an all-solid-state polymer-based IR sensor. Building on that platform, this work presents an optical sensor with substantially enhanced performance achieved through interfacial engineering. By strengthening the bond between the photothermal actuator (PDA NP/PDMS composite) and the piezoresistive laser-induced nanocarbon film via O_2_ plasma and silane coupling, and by integrating a flexible PDMS support, the resulting sensor exhibits a responsivity of 51.6 W^−1^ under 808 nm irradiation—an order-of-magnitude improvement over the unmodified device (2.2 W^−1^)—along with a 3.1 s response time. Wavelength-dependent characterization across 455–1550 nm reveals a photomechanical response whose responsivity correlates with the optical absorption coefficient of the PDA NP composite, decreasing from 93.1 W^−1^ at 455 nm to 14.4 W^−1^ at 1550 nm. Under blackbody radiation, the sensing mechanism transitions to a predominantly bolometric mode, consistent with the low absorption of PDA NPs at longer wavelengths. Using 455 nm as a representative case, the device further demonstrates stable sub-milliwatt detection and robust durability over extended cycling. These results confirm that interfacial reinforcement effectively enhances photomechanical transduction, while the observed wavelength-dependent behavior highlights the potential for tailoring spectral sensitivity through future engineering of photothermal materials toward broadband, cost-effective uncooled optical detection.

## Figures and Tables

**Table 1:** Specifications of the single-wavelength laser sources and the blackbody radiation source.

Wavelength	Model	Manufactory
455 nm	MLL III-455	Changchun Leishi Optoelectronic Technology Co., Ltd. (Changchun, China)
808 nm	L808P1000MM	Thorlabs Optoelectronics Technology (Shanghai) Co., Ltd. (Shanghai, China)
1550 nm	L1550G1	Thorlabs Optoelectronics Technology (Shanghai) Co., Ltd. (Shanghai, China)
Black body	CS500	DIAS Infrared GmbH (Dresden, Germany)

## Data Availability

The original contributions presented in this study are included in the article material. Further inquiries can be directed to the corresponding author.

## Abbreviations

The following abbreviations are used in this manuscript:

IR	Infrared
PDA NPs	Polydopamine Nanoparticles
PDMS	Polydimethylsiloxane
PI	Polyimide
DMA	Dynamic Mechanical Analysis
DMM	Digital Multimeter
MPTMS	3-mercaptopropyl trimethoxysilane
GPTMS	3-glycidyloxypropyl trimethoxysilane
SNR	Signal-to-noise ratio
NEP	Noise-equivalent power
